# Search in the Literature for Viral Hepatitis and Hepatocellular Carcinoma in Iran

**DOI:** 10.5812/hepatmon.9172

**Published:** 2013-04-29

**Authors:** Ali Kabir

**Affiliations:** 1 Department of Epidemiology, Faculty of Public Health, Shahid Beheshti University of Medical Sciences, Tehran, IR Iran

**Keywords:** Carcinoma, Hepatocellular, Hepatitis B, Hepatitis C, Iran, Epidemiology

Dear Editor,

I read with interest a nice paper by Zidan et al. about viral hepatitis as etiology of hepatocellular carcinoma in Iran and the world (1). They have presented useful information. However, maybe these comments can add some more information specifically about the Iranian literature. They have searched some databases. However, other useful sites like Scientific information Database (SID), Iranian Research Institute for Information Science and Technology (IRANDOC), Iranian Data Bank of Hepatitis Research and Iranian Data Bank of GI Cancer Research, are also available which first two ones are general scientific and two last sites are specific about gastroenterology. Scopus and Web of Science should also be considered. Although, these sites have overlap with mentioned databases in the paper; however, their importance is that they cover some parts of the literature which are not covered by other national or international databases. The importance of these sites is also because of Grey (unpublished/unavailable) literature. There are new systematic reviews and meta-analyses about epidemiology of HBV (2) and HCV (3) in general population and hemodialysis patients (4) of Iran which are available in Scopus and Web of Science; but, are not cited in such review article. These papers present better image from total prevalence of viral hepatitis in the country. At least the level of endemicity of the country has been shown more precisely and the knowledge gap in the field can also be determined. It seems that their search is not sufficiently thorough and comprehensive, at least about Iran.
